# Supplementary material for: Low-Toxicity Self-Photosensitized Biohybrid Systems for Enhanced Light-Driven H2 Production
Source: Int J Mol Sci. 2024 Mar 7;25(6):3085. doi: 10.3390/ijms25063085 (PMC10970202; doi:10.3390/ijms25063085)
Supplement: Supplementary file 1 [file ijms-25-03085-s001.zip › ijms-2848184-supplementary.pdf]

Supplementary Materials

Low-Toxicity Self-Photosensitized Biohybrid Systems for Enhanced Light-Driven H_2 Production

Yuelei Wang, Yuqi Liu, Long Bai, Jueyu Wang, Na Zhao *, Daizong Cui and Min Zhao *

College of Life Science, Northeast Forestry University, Harbin 150040, China; wangyuelei1020@163.com (Y.W.); liuyuqi0921@126.com (Y.L.); bailongs@126.com (L.B.); 18804503512@163.com (J.W.); siyu19831114@163.com (D.C.)

* Correspondence: bszhaona2013@163.com (N.Z.); 82191513@163.com (M.Z.)

Table S1. Ingredients of synthesizing CdS nanoparticles medium (Medium I) and light-driven hydrogen production medium (Medium II).[1]

Medium I		Medium II	
Ingredient	/L	Ingredient	/L
$Na_2HPO_4 \cdot 12\ H_2O$	17.1 g	$(NH4)_2HPO_4$	10 g
KH_2PO_4	3 g	K_2SO_4	2 g
NaCl	0.5 g	NaCl	0.3 g
NH_4Cl	1 g	$MgSO_4 \cdot 7\ H_2O$	0.2 g
1M $MgSO_4$	2 ml	$FeSO_4 \cdot 7\ H_2O$	4 mg
1M $CaCl_2$	0.1 ml	$ZnSO_4 \cdot 7\ H_2O$	0.9 mg
Yeast extract	0.5 g	$CuSO_4 \cdot 5\ H_2O$	0.4 mg
Tryptone	0.25 g	$MnSO_4 \cdot H_2O$	0.2 mg
Glucose	4 g	$CaCl_2 \cdot 2\ H_2O$	0.8 mg
		$Na_2B_4O_7 \cdot 10\ H_2O$	0.09 mg
		$Na_2SeO_3 \cdot 5\ H_2O$	0.6 mg
		$(NH_4)_6Mo_7O_{24}$	0.4 mg
		$(NH_4)_2Ni(SO_4)_2 \cdot 6\ H_2O$	0.9 mg
		Adjust pH to 7.0	
		Glucose	5.95 g

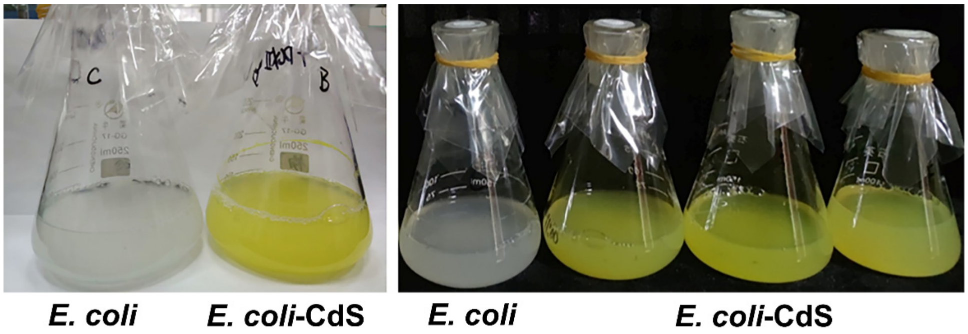

Figure S1. Photos of *E. coli* and *E. coli*-CdS hybrids.

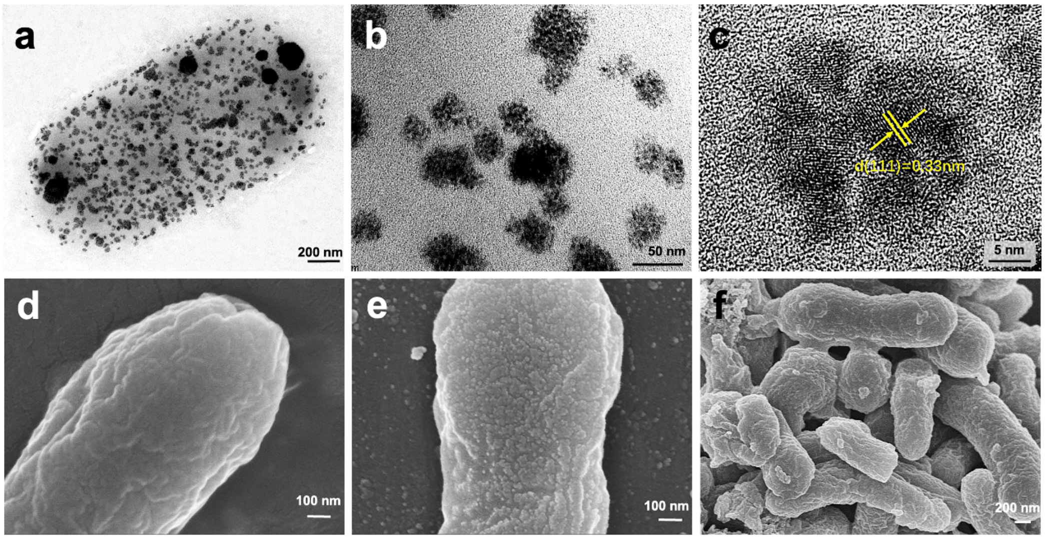

Figure S2. Characterization of *E. coli* and *E. coli*-CdS hybrids. (a, b, and c) TEM images of *E. coli*-CdS and biosynthesized CdS NPs. SEM images of *E. coli* (d) and *E. coli*-CdS hybrids (e and f).

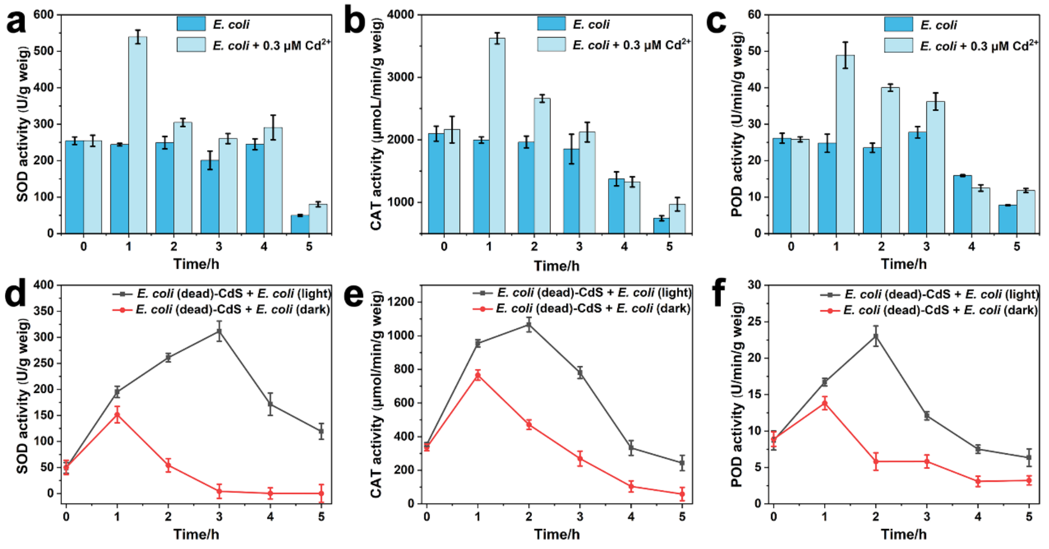

Figure S3. Toxicity assessment of Cd^{2+} and CdS NPs on whole bacterial cells. (a–c) Changes in SOD, CAT, and POD activities in *E. coli* cells exposed to 0.3 μM Cd^{2+} condition. (d–f) Effect of CdS NPs on SOD, CAT, and POD activities in *E. coli* (dead)-CdS hybrids under visible light illumination.

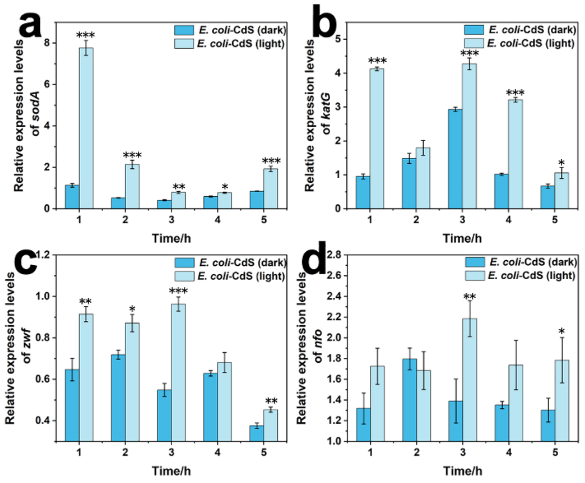

Figure S4. The relative expression levels of s*odA* (a), *katG* (b), *zwf* (c), and *nfo* (d) in the hybrid systems process with or without visible light irradiation.

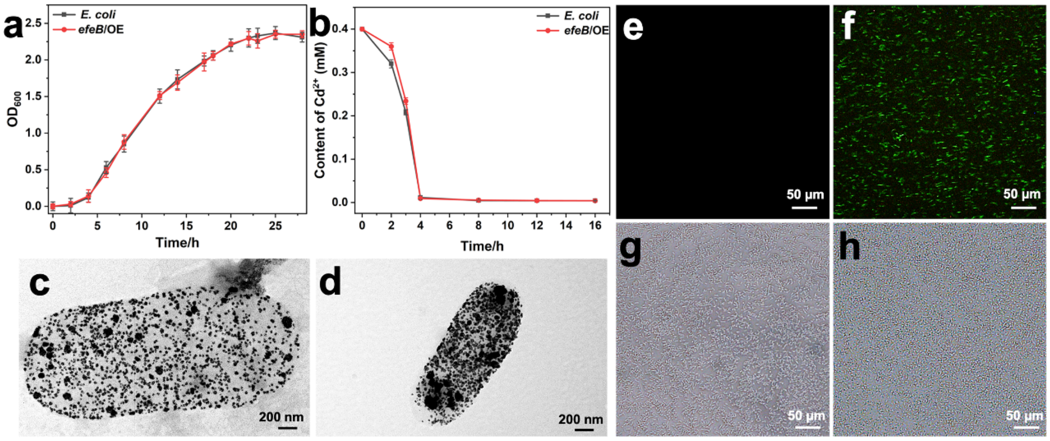

Figure S5. The effect of EfeB overexpression on *E. coli* activity. (a) Growth curves of *E. coli* and *efeB*/OE in aerobic LB medium. (b) During the synthesis of CdS NPs by *E. coli* and *efeB*/OE, suspensions were taken at different time points and the residual amount of cadmium ions in the culture medium was determined. (c and d) TEM images of the *efeB*/OE-CdS hybrid. The eGFP protein produces green fluorescence at 488 nm excitation wavelength (e and f). Bright field picture of *E. coli* (g) and *efeB*/OE (h).

Table S2. Concentration of MDA in *E. coli*-CdS and *efeB*/OE-CdS during 750 Wm^{-2} illumination.

Illumination time (h)	MDA (nmol g^{-1})	
	E. coli-CdS	*efeB*/OE-CdS
0	3.42 ± 0.12	3.41 ± 0.13
3	4.74 ± 0.81	1.99 ± 0.69
6	3.03 ± 0.35	0.56 ± 0.22
9	3.83 ± 0.27	2.79 ± 0.15
12	4.89 ± 0.71	2.49 ± 0.46

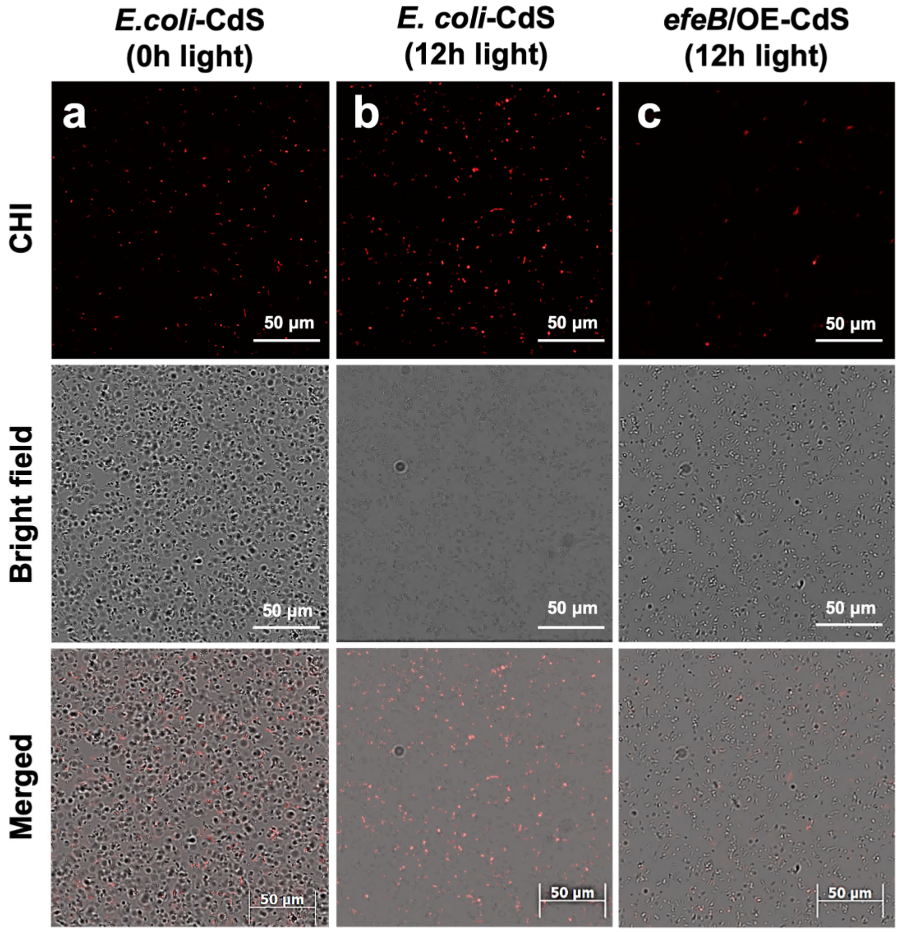

Figure S6. Accumulation of propidium iodide (PI) in hybrid systems cells. The cells of (a) *E. coli*-CdS (0 h), (b) *E. coli*-CdS (12 h), and (c) *efeB*/OE-CdS (12 h) hybrids were stained with PI after 12 h light illumination, using *E. coli*-CdS (0 h) as a control. The red fluorescence was observed by excitation at 546 nm wavelength.

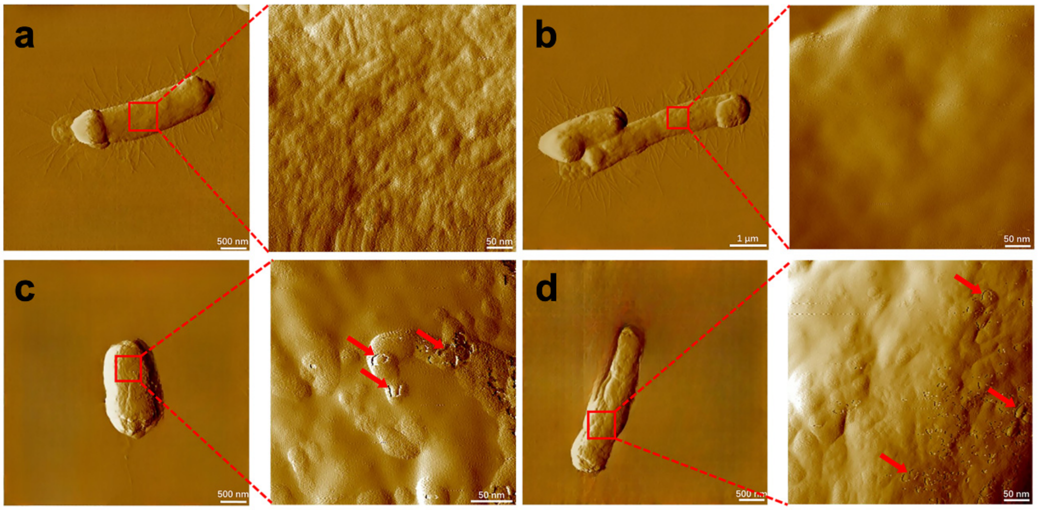

Figure S7. AFM study of the cell surface. AFM study of the surface topology of (a and b) *E. coli*, (c) *E. coli*-CdS (0 h), and (d) *efeB*/OE-CdS (0 h) cells without illumination. Magnified portion is ultrastructural images.

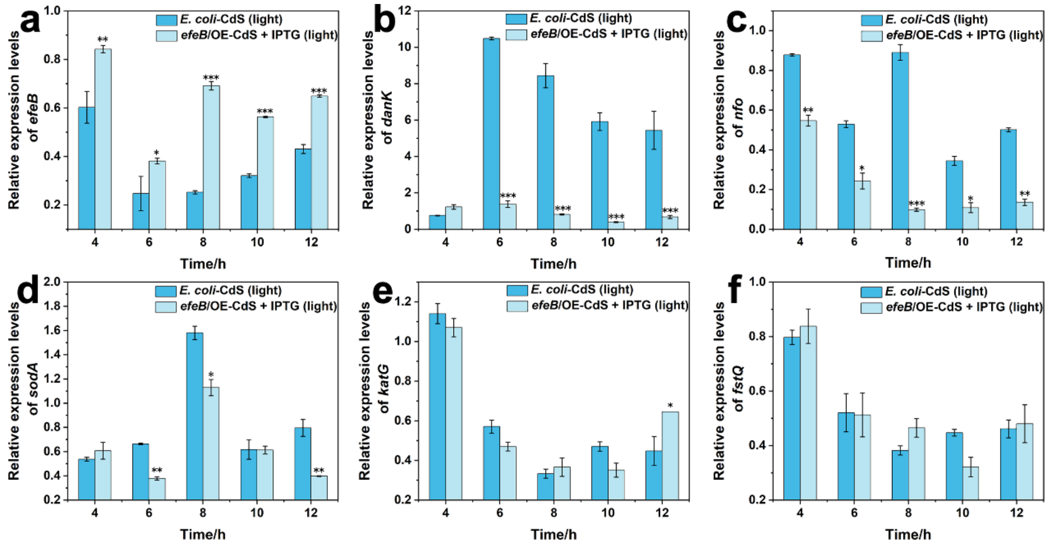

Figure S8. EfeB reduction mechanism of toxicity under illumination. The relative expression levels of *efeB* (a), *danK* (b), *nfo* (c), *sodA* (d), *katG* (e), and *fstQ* (f) in *E. coli*-CdS and *efeB*/OE-CdS during light-driven hydrogen production.

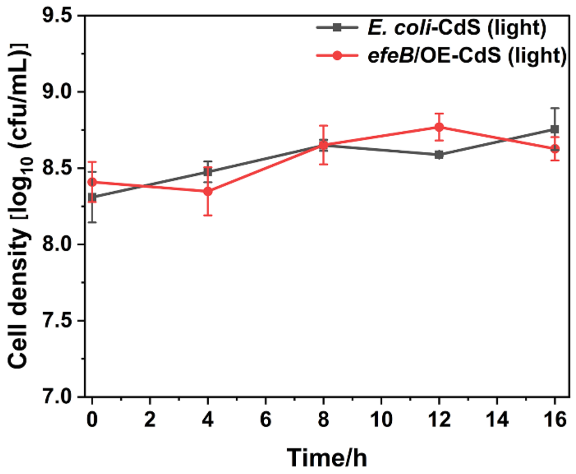

Figure S9. Viability of the hybrid system during the hydrogen production process with visible light irradiation.

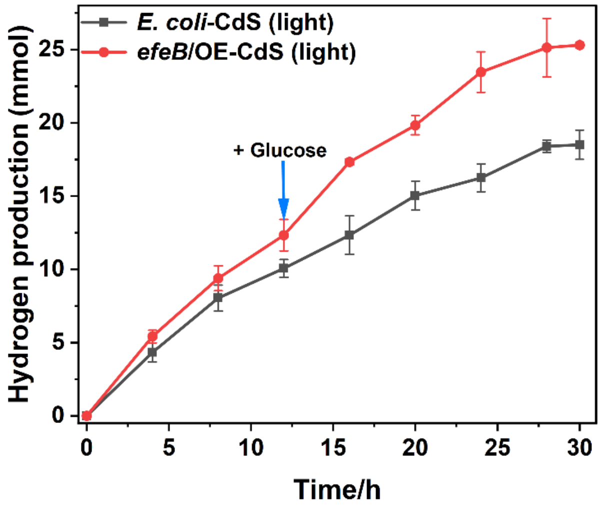

Figure S10. After glucose supplementation to a final concertation of 6 g /L at 12 h, hydrogen production by the *efeB*/OE-CdS photosynthetic system and control experiments.

Table S3. Apparent quantum efficiency of biohybrid systems compared with others reported biotic-abiotic biohybrids.

Biotic-abiotic biohybrid	Wavelength (nm)	Application	AQE (%)	Reference
M. thermoacetica–gold nanoclusters	405	CO_2 reduction	2.86	[2]
M. barkeri- CdS	395	CO_2 reduction	0.34	[3]
T. denitrificans-CdS	400	Denitrification	2	[4]
E. coli-TiO_2/ methylviologen	420	H_2 production	0.1	[5]
E. coli-$AglnS_2/In_2S_3$	720	H_2 production	3.3	[6]
D. desulfuricans-CdS	445	H_2 production	4	[7]
E. coli-CdS	420	H_2 production	0.1	[8]
E. coli-CdS	420	H_2 production	4.16	This work
efeB/OE-CdS	420	H_2 production	5.94	This work

Table S4. Primers used in this study.

Genes	Primers	Sequences
efeB	G-efeB-F	5'-CGGAATTCATGCAGTATAAAGATGAAAACGG-3'
	G-efeB-R	5'-CCCAAGCTTTTAAACCCGCAATAACGC-3'
	RT-efeB-F	5'-CGGTTGTTTCGCTTGTTG-3'
	RT-efeB-R	5'-TTATCGGGCGCAATGTAG-3'
ftsQ	RT-ftsQ-F	5'-CGAAACAGCGAAGAAGAGG-3'
	RT-ftsQ-R	5'-ATCCAGCCCAACACGAC-3'
katG	RT-katG-F	5'-TGGAAAGCGACCGACGAATC-3'
	RT-katG-R	5'-CCGCACGCAGGACGGAGTTA-3'
sodA	RT-sodA-F	5'-GCAACAACGCTGGCGGTCAC-3'
	RT-sodA-R	5'-TATCAACGGAGCCGAAGTCA-3'
nfo	RT-nfo-F	5'-GGACATCCGGTCACTGAAGC-3'
	RT-nfo-R	5'-AGGGTGGAAGTTGAGCAAAGAA-3'
zwf	RT-zwf-F	5'-CGAACAAGAGCAGCAATACAG-3'
	RT-zwf-R	5'-TACGCAGGTAGAATGGCACA-3'
dnaK	RT-dnaK-F	5'-GCTGTTCAGGGTGGTGTTC-3'
	RT-dnaK-R	5'-GTGCTTGGTCGGGATAGTG-3'
16s rDNA	RT-16s-F	5'-CCTACGGGAGGCAGCAG-3'
	RT-16s-R	5'-ATTACCGCGGCTGCTGG-3'

References

1. Wang, B.; Zeng, C.; Chu, K.H.; Wu, D.; Yip, H.Y.; Ye, L.; Wong, P.K. Enhanced Biological Hydrogen Production from *Escherichia coli* with Surface Precipitated Cadmium Sulfide Nanoparticles. *Adv. Energy Mater.* **2017**, *7*, 1700611. doi:https://doi.org/10.1002/aenm.201700611.
2. Zhang, H.; Liu, H.; Tian, Z.; Lu, D.; Yu, Y.; Cestellos-Blanco, S.; Sakimoto, K.K.; Yang, P. Bacteria photosensitized by intracellular gold nanoclusters for solar fuel production. *Nat. Nanotechnol.* **2018**, *13*, 900-905. doi:https://doi.org/10.1038/s41565-018-0267-z.
3. Ye, J.; Yu, J.; Zhang, Y.Y.; Chen, M.; Liu, X.; Zhou, S.G.; He, Z. Light-driven carbon dioxide reduction to methane by *Methanosarcina barkeri*-CdS biohybrid. *Appl. Catal., B* **2019**, *257*. doi:https://doi.org/10.1016/j.apcatb.2019.117916.
4. Chen, M.; Zhou, X.-F.; Yu, Y.-Q.; Liu, X.; Zeng, R.J.-X.; Zhou, S.-G.; He, Z. Light-driven nitrous oxide production via autotrophic denitrification by self-photosensitized *Thiobacillus denitrificans*. *Environ. Int.* **2019**, *127*, 353-360. doi:https://doi.org/10.1016/j.envint.2019.03.045.
5. Honda, Y.; Watanabe, M.; Hagiwara, H.; Ida, S.; Ishihara, T. Inorganic/whole-cell biohybrid photocatalyst for highly efficient hydrogen production from water. *Appl. Catal. B-Environ.* **2017**, *210*, 400-406. doi:https://doi.org/10.1016/j.apcatb.2017.04.015.
6. Jiang, Z.F.; Wang, B.; Yu, J.C.; Wang, J.F.; An, T.C.; Zhao, H.J.; Li, H.M.; Yuan, S.Q.; Wong, P.K. AglnS$_2$/In$_2$S$_3$ heterostructure sensitization of *Escherichia coli*

for sustainable hydrogen production. *Nano Energy* **2018**, *46*, 234-240. doi:https://doi.org/10.1016/j.nanoen.2018.02.001.

7. Martins, M.; Toste, C.; Pereira, I.A.C. Enhanced Light-Driven Hydrogen Production by Self-Photosensitized Biohybrid Systems. *Angew. Chem. Int. Ed.* **2021**, *60*, 9055-9062. doi:https://doi.org/10.1002/anie.202016960.

8. Honda, Y.; Shinohara, Y.; Watanabe, M.; Ishihara, T.; Fujii, H. Photo-biohydrogen Production by Photosensitization with Biologically Precipitated Cadmium Sulfide in Hydrogen-Forming Recombinant *Escherichia coli*. *ChemBioChem* **2020**, *21*, 3389-3397. doi: https://doi.org/10.1002/cbic.202000383.
